# Health Care Service Readiness and Quality of Care for Sexual Violence in Garissa, Kwale, Narok, and West Pokot Counties, Kenya: A Mixed-Methods Study

**DOI:** 10.9745/GHSP-D-24-00344

**Published:** 2026-08-10

**Authors:** Julius Njogu, Lydiah Ndung’u, Harmon Momanyi, Monicah Nthumbi, Alberta Wambua, Halima Zaid, Charlotte Pahe, Francis Gwama, Alison Malmqvist, Claire W. Rothschild

**Affiliations:** a Population Services International, Nairobi, Kenya.; b Population Services Kenya, Nairobi, Kenya.; c Gender Violence Recovery Centre, Nairobi, Kenya.; d Embassy of Denmark in Kenya, Nairobi, Kenya.; e Ministry of Health, Kwale County, Kwale, Kenya.; f Population Services International, Washington DC, USA.

## Abstract

**Background:**

The health care system is a critical entry point for sexual violence response including care and linkages to auxiliary services. However, detailed data on quality and readiness of facility-based sexual violence care in African settings is sparse, hindering measurement and improvements.

**Methods:**

We report results from the first wave of longitudinal mixed-methods study conducted between July and September 2022 to assess readiness and quality of sexual violence services in 4 counties in Kenya supported by the *Accelerate* program. Health facility assessments were conducted within all (N=123) program-supported facilities in the 4 counties, including provider interviews; visual inspection and audit of essential infrastructure and commodities; and chart abstraction. We computed descriptive data summaries, and in-depth interviews from 40 purposively selected providers were analyzed thematically.

**Results:**

Of 123 study facilities, 54% had a provider who received GBV in-service training in the past 12 months. Most facilities (85%) routinely offered GBV care. Of facilities offering GBV care, most stocked antibiotic for sexually transmitted infections (STIs) (71%), any emergency contraception pill (ECP) option (88%), and tetanus vaccine (93%), and most provided GBV counseling (90%). However, availability of hepatitis B vaccine was low across facility tiers (14%–25%). Dispensaries/clinics documented low availability of HIV post-exposure prophylaxis (PEP) for children and adults (20% and 47%, respectively), SGBV register (20%), and post-rape care forms (27%). Of 285 abstracted charts, most were in hospitals (62%) and for survivors under 18 years (69%). Just 59% of survivors presented promptly, within 3 days, for health care services. Of survivors who were eligible for care, a considerable proportion missed psychosocial assessment (32%), PEP for HIV (22%), ECP (17%), and treatment for bacterial STIs (15%). Qualitative data revealed service delivery gaps driven by disruptions in supplies and provider capacity gaps. Many providers indicated concerns in the chain of evidence due to gaps in documentation and logistical support for health care providers when providing legal testimony in courts.

**Conclusions:**

Readiness to provide a minimum care package for sexual violence was low, with steep gradients across facility tiers. Delivery of timely and quality-assured sexual violence care requires addressing structural gaps driven by stock-out of supplies and inadequate providers’ support including training. These corrective actions should be augmented by community-based interventions that link survivors to health care.

## Abstract (precis)

Sexual violence survivors in surveyed counties in Kenya face a dual barrier to effective care: community-level obstacles—including stigma, lack of awareness, and reliance on informal justice systems—delay health facility access beyond the critical 72-hour window, while health system gaps prevent those who do seek care from receiving complete, quality-assured care and treatment.

## Teaser

**Key Findings:**

- Although per policy all tiers of the Kenyan health care system should be able to provide an essential minimum package of services to survivors of gender-based violence (GBV), we found low prevalence of facility readiness, with the largest gaps at the lowest levels of the health care system (dispensaries and health centers).
- A considerable fraction of sexual violence survivors missed opportunities for medical care, driven by late presentation at health facilities, making survivors ineligible for specific medical treatment, or by providers’ failure to initiate medical treatment such as emergency contraception, HIV post-exposure prophylaxis, presumptive treatment of bacterial STIs, and psychosocial care for trauma recovery.

**Key Implications:**

- The widespread lack of availability of a minimum package of essential GBV services—particularly in lower-tier health facilities closest to the community—means that GBV survivors presenting for care are often not offered time-sensitive post-exposure medical treatment.
- Kenyan policymakers and program implementers should focus on strengthening GBV-related supplies, infrastructure, provider and facility capacity for treatment, and effective referral structures to reduce barriers to timely and effective care for survivors of GBV.

## BACKGROUND

Sexual violence impacts about one-third of women globally, with a multitude of negative consequences to health and well-being, including morbidity and mortality from injuries, sexually transmitted infections (STIs), unwanted pregnancy, and psychological trauma, among other effects.^1^ Best-practice standards in care and treatment for sexual violence have underscored the importance of a multisectoral approach that provides coordinated, timely, and comprehensive services that are responsive to the needs of survivors, including medical care, lifesaving treatments, and effectively linking survivors with non-clinical resources.^2^^–^^4^ At its best, the health care system can serve as a safe space and critical entry point to care and justice for survivors of sexual violence.^3^^,^^5^ Evidence indicates health care providers are well-placed to act as first responders to proactively identify survivors and deliver and coordinate integrated medical care, and often are also best positioned to connect survivors to a constellation of non-medical services such as psychosocial support and legal aid.^6^^,^^7^

Enabling health care providers to be effective in a frontline care coordination role requires the structural support of health care systems, including definition of a basic package of essential sexual violence services provided by health facilities; articulation of pragmatic approaches to violence screening and active case identification; and ongoing training and supervision supported by clinical protocols.^8^ The health care system should also ensure continuous availability of essential clinical supplies, commodities, equipment, and infrastructure; straightforward referral pathways; and documentation and routinized monitoring and evaluation for adaptive management, quality improvement, and evidence-based decision making at national and sub-national levels.^8^

Despite the key role the health care system can and should play in supporting survivors of sexual violence, many health facilities in low- and middle-income country (LMIC) settings remain under-resourced to deliver effective care.^9^^,^^10^ While integrated service delivery models and specialized sexual and gender-based violence (SGBV) in-service trainings have been implemented within health facility settings in a number of LMIC settings, case studies from various countries have identified common barriers to effective SGBV care responses.^9^ These include high clinical staff turnover; inefficient and inadequate cascading of training to frontline health care providers; and insufficient infrastructure to ensure survivors’ privacy within health care settings.^9^ Deeper understanding of care pathways and the current quality and availability of sexual violence services within LMIC health care systems are critical for identifying gaps and developing effective health systems-focused interventions that integrate global best practice and context-specific capacity.^7^^,^^11^

Evidence from Kenya suggests numerous barriers to high-quality, person-centered, and comprehensive care for survivors of sexual violence. Widespread stigmatization of care-seeking and reliance on traditional structures of redress, combined with poverty and low awareness and availability of quality post-violence support services, result in nonexistent care-seeking and low receipt of critical preventative care and treatment.^12^^–^^15^ While there is a dearth of published data on the quality of routine health care provided to survivors of sexual violence in Kenya, a recent study in two urban public hospitals revealed considerable gaps in sexual violence case management, including failures to provide treatment to prevent pregnancy, HIV, and other STIs and inadequate clinical examination and documentation of the care process, including findings of the medical examination and tests.^16^

To align with the global commitments to the International Conference on Population and Development (ICPD+25), in 2021 Kenya made 12 bold promises to prevent, respond to, and eventually eliminate all forms of GBV including sexual violence by 2026.^17^ In June 2022, the Kenyan Ministry of Health (MOH) implemented several countrywide initiatives to strengthen responses to GBV at all tiers of the health care system. Among these activities included the launch of a series of updated policy documents, training manuals, and reporting tools related to sexual and reproductive health and rights (SRHR) service delivery and data reporting.^18^ Specific to GBV response in health care settings, the MOH disseminated guidelines on management of intimate partner violence, forensic management of SGBV, and a training manual on female genital mutilation prevention and management of health complications. In addition, a revised national GBV register was disseminated to integrate reporting of all forms of violence at the facility level including sexual violence, intimate partner violence, physical violence, and harmful practices such as marriages of children below the age of 18 and female genital mutilation. Following introduction of the revised policies and tools, a national in-service training in GBV case-management was cascaded to the national and sub-national (county) health workforce through a trainer-of-trainers model.

GBV remains a pressing public health concern in Kenya. The 2022 Kenya Demographic and Health Survey estimated that 13% of women aged 15–49 had ever experienced sexual violence, with 7% reporting sexual violence in the past 12 months.^19^ To address the GBV problem, the *Accelerate* program (2021–2026), funded by the Embassy of Denmark in Kenya, is being implemented in 13 underserved counties. The *Accelerate* program aims to accelerate progress toward realization of ICPD+25 promises of eliminating preventable maternal mortality, unmet need for contraceptive information and services, and GBV and harmful practices.^20^ Implemented by Population Services Kenya and the Gender Violence Recovery Center, with research, monitoring, and evaluation conducted by Population Services International, the *Accelerate* program includes integrated supply- and demand-side SRHR activities. The program partners with 425 public and private health facilities in implementation counties to increase facility readiness to deliver quality-assured and integrated SRHR and GBV care. *Accelerate* also conducts social and behavior change activities aimed to increase awareness and knowledge of rights and shift individual attitudes and social and community norms among women, male partners, and community leaders.

In this article, we report findings from the first wave of health facility assessments and provider interviews conducted within *Accelerate*-supported facilities in 4 counties to characterize experiences from the program’s early implementation period. We conducted a concurrent mixed-methods study to assess health facility readiness and quality of care provided to survivors of sexual violence, as well as provider perspectives associated with facility-based care services.

## METHODS

### Study Setting

In 2013, Kenya devolved the health care system from a single central government to 47 semi-autonomous units of government known as counties.^21^ Health functions are delineated with the central government providing policy and regulation roles, while the service delivery functions, including planning, budgeting, and implementation including partners coordination, human resource management, and capacity development, were decentralized to counties. Both private and public health facilities are structured into a 6-tiered system, hierarchically organized based on scope and complexity of services provided.^22^ The first tier comprises community health units that provide preventive and promotive health services. Tiers 2 and 3 encompass primary health care facilities including dispensaries/clinics and health centers, respectively. Dispensaries/clinics are mandated to provide health promotion and prevention services, as well as basic outpatient services including laboratory services,^23^ while health centers provide an expanded range of comprehensive services such as rehabilitative and basic surgical services. Tiers 4 and 5 represent sub-county and county hospitals that serve as primary and secondary referral centers, respectively, offering comprehensive and specialized health services. Tier 6 comprises national hospitals offering specialized health services exclusively and serve as tertiary referral facilities.

According to the national sexual violence management guidelines, a rape survivor presenting within 72 hours at a health facility should receive a minimum package of care (Table 1).^24^ This minimum care package includes 4 major components. The first component includes collection and documentation of survivors’ information through history-taking, medical examination, administration of laboratory tests, and collection and storage of forensic specimens. Specific laboratory services included in the essential minimum package include a battery of tests to assess STIs such as an HIV test, syphilis test, urinalysis, and hepatis B and C tests. Hemoglobin, creatinine, and alanine aminotransferase tests are also required to establish a baseline value prior to administration of HIV post-exposure prophylaxis (PEP). Other essential tests include a pregnancy test, high vaginal swab (HVS), and oral/anal-rectal swabs critical for collection of forensic evidence such as spermatozoa. The second component of the minimum care package is administration of emergency prophylactic and preventative treatments such as PEP with antiretroviral drugs, the emergency contraceptive pill, STI prophylaxis, and hepatitis B and tetanus vaccine provision. The third component involves counseling services to address traumatic experiences and adherence to care, while the fourth entails linkage to other services including HIV care, psychological support, access to justice, and rescue to a safe shelter facility.

**TABLE 1.** Minimum Care Package^a^ for Rape Survivors Presenting in a Health Facility Within 72 Hours, Kenya

**Component**	**Specific Services**
History, examination, diagnostic tests, and sample collection	Obtaining informed consent Taking history and medical exam Laboratory tests including HIV, pregnancy diagnostic test, hemoglobin, syphilis test (also known as venereal disease research laboratory), hepatitis B virus test, hepatitis C test, creatinine test, alanine aminotransferase test, and urinalysis Collection of forensic samples including high vaginal swab, oral/anal-rectal swabs, hairs, semen, and blood-stained clothes Appropriate labeling, packing, and storage of samples
Assess eligibility and provide emergency medical interventions	Post-exposure HIV prophylaxis with antiretroviral drugs Administration of emergency contraceptive pills within 120 hours for pregnancy prevention: either levonorgestrel tablets (e.g., Postinor 2 tablets start) or combined oral contraceptive pills (e.g., Microgynon 4 tablets start or Nordette 8 tablets start) STI prevention and treatment with the recommended antibiotics and antifungals Prophylaxis hepatitis B vaccination Prophylaxis tetanus toxoid injection
Counselling and support	Trauma counseling Pre- and post-HIV test counseling Adherence counseling
Referral and linkage to other services	HIV clinic care Psychosocial support Police and legal care Shelters

^a^ Adapted from national guidelines on management of sexual violence.

### Study Sites

The study was conducted in 4 underserved rural counties including Garissa, Kwale, Narok, and West-Pokot. Study counties were purposively selected from the 13 *Accelerate*-supported counties as “learning lab” counties for the project that represented the large cultural, socioeconomic, and geographic diversity of the implementation area (Figure). A subset of health facilities within each *Accelerate*-supported sub-county was selected to receive program support through a co-creation process conducted by the *Accelerate* program implementation team and each respective county’s health program managers, based on relative burden of GBV and unmet need for SRHR services in the catchment area. For the purposes of this study, the research team sampled and recruited all 123 program-supported health facilities in the study counties. The research team was not directly involved in the selection of which facilities received *Accelerate* program support.

**FIGURE:**
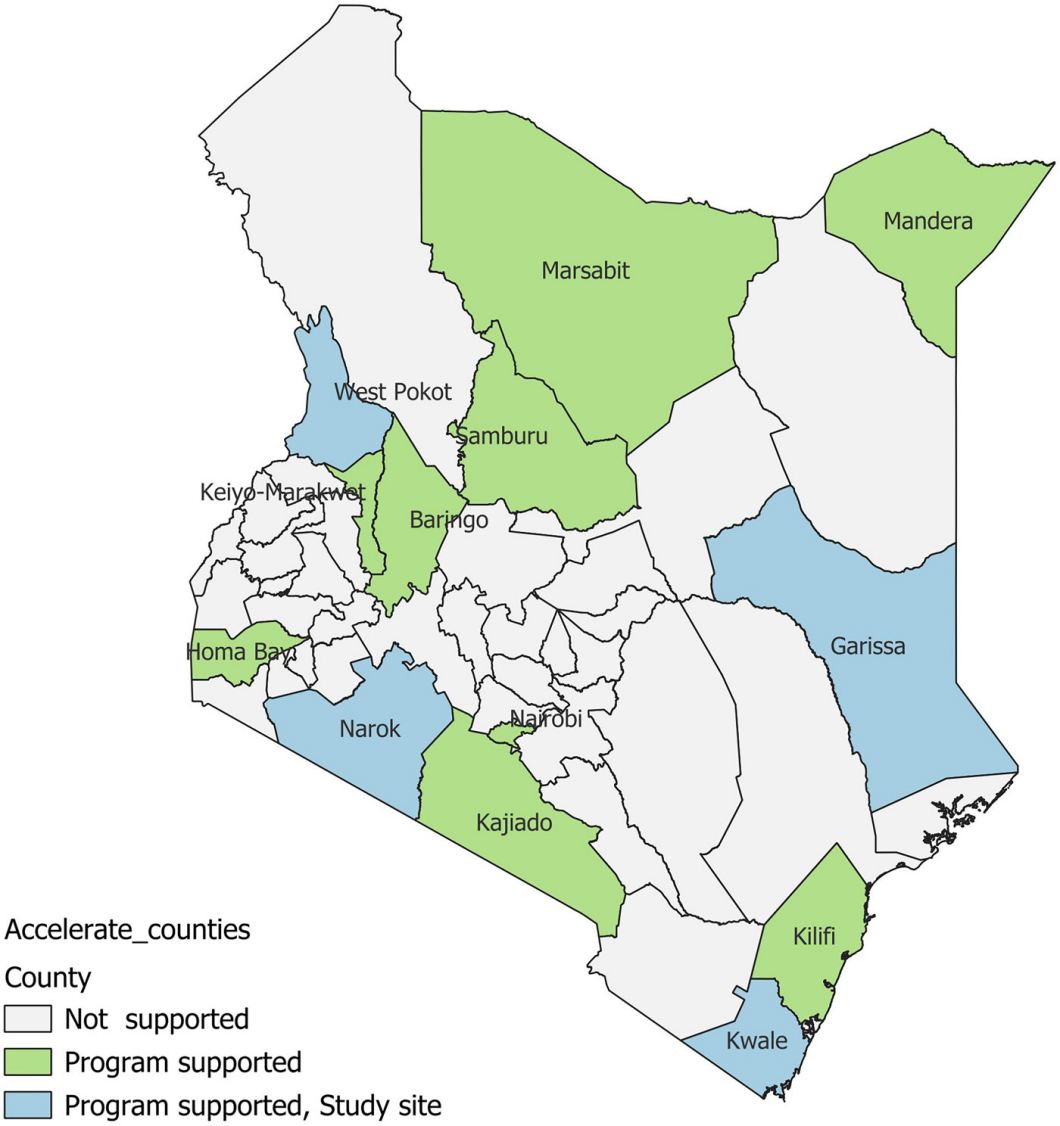
Scope of Accelerate Program-Supported Counties in Kenya

### Study Design

This analysis draws from an ongoing longitudinal study that uses a concurrent mixed-methods design, with health facility data collection planned annually for 3 years representing the early, mid, and late implementation periods of the *Accelerate* program. In this article, we report on data collected from the first wave of data collection, conducted between July and September 2022, representing the early implementation period. By the time of data collection, study facilities had received limited program support due to delays related to co-creation activities with the stakeholders and program recipients. Therefore, presented data describe early implementation experiences rather than baseline results.

### Data Collection

Quantitative data on facility readiness and quality of care were collected through health facility assessments, which included interviews with in-charge providers on facility service delivery and infrastructure; visual inspection and audit of essential infrastructure, commodities, and supplies; and structured chart abstraction. The in-charge interviews and visual inspection components assessed service readiness to deliver sexual violence care by capturing data on the availability of clinical care and treatment, including laboratory capacity and availability of relevant equipment, supplies, and commodities; forensic medical examinations and reporting capacity; basic infrastructure requirements such as private examination areas; and clinical guidelines, job aids, and informational, educational, and communication (IEC) materials. Data were also gathered related to the providers’ exposure to in-service GBV training in the past 12 months preceding the survey date.

In a subset of study health facilities, a semi-structured in-depth interview (IDI) guide was administered to explore provider knowledge, experiences, and attitudes related to clinical management of sexual violence. Forty IDIs were completed among purposively sampled providers by county, facility tier, and public-private sector. We interviewed one provider per facility that was identified by the facility in-charge as the most experienced in provision of GBV services. Interviews were conducted in English by trained field researchers. In Kenya, English is the primary instructional language in which all medical and nursing professionals are expected to have a relatively high level of proficiency. Interviews were conducted in a private space in the provider’s facility and audio-recorded with verbal consent of the participating providers.

Within all sampled health facilities, a retrospective review with structured abstraction of clinical charts was completed to describe timeliness of care-seeking and assess documented quality of care provided to survivors of sexual violence. Charts were selected for extraction if the date of facility service was within 6 months of the assessment date; if more than 30 charts were available at a particular facility, we selected the 30 charts with the most recent service date. No charts were abstracted if the facility did not have documented cases available in the 6-month review period. A structured data form was deployed to abstract survivors’ socio-demographic data, type of violence and aggressor, violence reporting, and administered services including examination, laboratory tests, treatments, and psychosocial services. The data abstraction form was adapted to mirror the national SGBV register ^24^ and pretested prior to finalization. Data were systematically extracted from the facility SGBV register. We cross-checked the register and post-rape care (PRC) form to enter any information that was available on the survivor from either data source.^24^ In instances where there was conflicting information by data source, we used data from the PRC form, as it captures comprehensive information about the survivor and provided care. No individually identifiable data were collected during abstraction or during the assessment. Abstracted data were used to determine quality of care provision by assessing the proportion of charts that documented provision of specific care components for which the survivor was eligible per the national guidelines (Table 2).^24^

**TABLE 2.** Study Outcomes, Definitions, and Data Sources

**Outcome Variable**	**Study Definition** ^a^	**Data Source**
Functional laboratory service	A health facility with a laboratory that was able to offer any medical testing service on the date of the assessment	Health facility assessment
Psychosocial assessment completed	Survivors who needed psychosocial assessment with the following attributes in their chart: Survivors presenting for an initial health care visit in a study health facility Presented with a history of any form of sexual violence.	Chart reviews
Post-exposure HIV prophylaxis administered	Survivors who needed post-exposure prophylaxis with antiretroviral drugs with the following attributes in their chart: Survivors presenting for an initial health care visit in a study health facility Health facility visit was within 3 days after sexual violence Presented with a history of significant risk for HIV infection in the event of rape, defilement, or other forms of penetrative sexual violence Absence of HIV positive status (i.e., survivor tested negative for HIV or was not documented as a known HIV-positive individual)	Chart reviews
STI treatment administered	Survivors who needed prophylaxis and treatment with antibiotics to manage STIs with the following attributes in their chart: Survivors presenting for an initial health care visit in a study health facility Health facility visit was within 3 days after sexual violence Presented with a history of significant risk for STI infection in the event of rape, defilement, or other forms of penetrative sexual violence	Chart reviews
Emergency contraception pill administered	Survivors who needed emergency contraception pills with the following attributes in their chart: Survivors presenting for an initial health care visit in a study health facility Health facility visit was within 5 days after sexual violence Female survivors of reproductive age (15–49 years) History of penetrative vaginal sexual violence by a male perpetrator Absence of pregnancy (i.e., survivor tested negative for pregnancy or was not documented as being pregnant)	Chart reviews

^a^ While national guidelines specified eligibility for prophylaxis and treatment in hours, we converted this to an equivalent duration in days based on dates and times provided in the charts.

### Data Analysis

We present descriptive statistics for categorical measures including summaries of counts and proportions of health facility characteristics, service readiness, timelines, and quality of care provided to survivors. We also report on measures of central tendencies for continuous variables (means and standard deviation [SD]; median and interquartile range [IQR]). Missing values are presented in descriptive summaries rather than imputation, as missing values for data sources such as clinical chart reviews are informative to assess quality and completeness of documentation. We present descriptive comparisons across tiers of the health care system. All analyses were computed using Stata 15.1 (StataCorp, College Station, TX).

Qualitative interviews were transcribed verbatim under the supervision of the second and third authors (LN and HM). Transcribed data were compared with the audio files to address quality concerns prior to analysis. Reviewed transcripts were imported to Dedoose, a cloud-based application for managing and analyzing qualitative data. A hybrid approach of inductive and deductive coding was used to analyze the data thematically.^25^ An initial codebook was developed using predefined codes from the interview guide. All transcripts were read multiple times by 2 expert qualitative data analysts (MS and JO) to familiarize themselves with the interview content and identify emerging codes and sub-codes for documentation in the final coding structure. Both analysts worked collaboratively to ensure high inter-coder reliability. Each interview was reviewed and coded, with relevant excerpts labeled with respective codes and sub-codes. Dedoose qualitative analysis software was used to review data patterns and identify recurrent themes. Qualitative analysis was stratified by type of facility to determine if perspectives differed by tiers of the health care system. We present relevant qualitative findings to complement and explain the quantitative analysis. De-identified excerpts are reported to characterize analyzed themes.

### Ethical Considerations

All study procedures were reviewed and approved by the AMREF Health Africa in Kenya Ethics and Scientific Review Committee (P1168-2022). Authorization to conduct research was obtained from the Director of Health at each study county. Verbal informed consent was provided by health facility in-charges prior to data collection. Providers who participated in health facility assessment and IDIs also verbally consented.

## RESULTS

### Interviewed Sample

All 123 *Accelerate*-supported health facilities consented to participate in the study, including 86 dispensaries/clinics (Tier 2), 24 health centers (Tier 3), and 13 hospitals (Tiers 4 and 5) (Table 3). Thirty-nine percent of the study facilities were located in Kwale county, and the remaining 61% were split across Garissa, Narok, and West Pokot (19%, 20%, and 22%, respectively). Forty providers completed IDIs, including 11 dispensary/clinic providers, 17 health center providers, and 12 hospital providers; most providers were based in public health facilities (75%). Of these providers, the majority were nurses (70%) and female (58%). The mean age of interviewed providers was 36.7 years (SD=8.7) and with a similar median (32 years; IQR: 28–40).

**TABLE 3.** Availability of GBV-Related Infrastructure and Trained Staff by Tier of Care, Kenya, No. (%)

**Characteristics**	**Dispensary/Clinic (Tier 2)** **N=86**	**Health Center** **(Tier 3)** **N=24**	**Hospital** **(Tiers 4 and 5)** **N=13**	**Total ** **(All Tiers)** **N=123**
**County name **				
Garissa	11 (12.8)	8 (33.3)	4 (30.8)	23 (18.7)
Kwale	41 (47.7)	4 (16.7)	3 (23.1)	48 (39.0)
Narok	11 (12.8)	10 (41.7)	4 (30.8)	25 (20.3)
West Pokot	23 (26.7)	2 (8.3)	2 (15.4)	27 (22.0)
**Has routine GBV care services**	71 (82.6)	22 (91.7)	12 (92.3)	105 (85.4)
**At least 1 health facility staff received in-service training in the past 12 months **				
Any GBV case-management training	40 (46.5)	17 (70.8)	9 (69.2)	66 (53.7)
Courtroom and forensic medical training	6 (7.0)	4 (16.7)	5 (38.5)	15 (12.2)
**Availability of a private consultation/examination room for confidential services **				
Auditory and visual privacy	76 (88.4)	22 (91.7)	13 (100)	111 (90.2)
Auditory privacy only	1 (1.2)	0 (0)	0 (0)	1 (0.8)
Visual privacy only	7 (8.1)	1 (4.2)	0 (0)	8 (6.5)
No privacy	2 (2.3)	1 (4.2)	0 (0)	3 (2.4)
**Availability of functional laboratory services on the day of the assessment **				
Yes	40 (46.5)	22 (91.7)	12 (92.3)	74 (60.2)
No laboratory services at the time	10 (11.6)	1 (4.2)	0 (0)	11 (8.9)
No, does not have a laboratory	36 (41.9)	1 (4.2)	1 (7.7)	38 (30.9)

Abbreviation: GBV, gender-based violence.

### Availability of GBV Infrastructure and Coverage of Trained Providers

Most facilities had a consultation or examination room that ensured audio and visual privacy for confidential GBV services (90%) (Table 3). Sixty percent of facilities had functional laboratory services on the date of the assessment. Nearly all hospitals (92%) had functional laboratory services compared to fewer than one-half (47%) of dispensaries/clinics. Approximately half (54%) of facilities had at least 1 staff who had attended an in-service GBV training in the past 12 months, with higher prevalence of recently trained staff at health centers and hospitals (69% or more) compared with dispensaries/clinics (47%). Only 12% of all facilities had any staff who had received GBV training that was focused on forensic medicine and legal skills (dispensaries/clinics, 7%; health centers, 17%; hospitals, 39%). Similarly, qualitative interviews indicated limited coverage of in-service GBV trainings, causing competency gaps when trained providers left or were transferred to another facility. More than one-half of primary health care providers, in Tiers 2 and 3, reported a complete lack of GBV-trained providers in their facilities. Several primary health care providers reported feeling “pressured” when managing survivors because they were not conversant with the current clinical standards.

*… Nobody has been trained on GBV in our facility. We are just trying to work with what we were trained from college*. – Health Center 1, County B, Female Clinical Officer

*… it’s not adequate because we need to train as many service providers as possible. So that we don’t have a vacuum whenever one person leaves.* – Hospital 2, County C, Male Nurse

In addition to clinical training, providers reported concerns related to inadequate training on interpersonal/psychosocial care and legal testimony. Interviewed providers stated their belief that many providers feel nervous and inadequate and that they lack confidence when presenting medico-legal evidence in the courtroom due to lack of legal and GBV clinical skills.

*… Most of the health care workers are afraid of attending these cases in courts, but once taken through the training you see it is very easy.* – Health Center 2, County B, Male Clinical Officer

Another provider felt health care providers should be trained on the management of sexual violence related to minors including effective strategies to mitigate provider–survivor communication challenges among the young survivors.

*… mostly especially in handling children below the age of 10, I think we do require training … it requires some sort of special approach, because they don’t really tell you exactly what happened … there is a communication barrier usually there …* – Hospital 2, County B, Female Doctor

### Readiness to Provide Minimum Care Package Components for Sexual Violence

Most facilities in the sample reported offering routine GBV case-management services (85%) (Table 3). Of facilities offering GBV care (n=105/123), just 39% had PRC forms, a prerequisite tool for documenting medico-legal evidence, and 33% were currently documenting cases using the national SGBV register (Table 4). None of the facilities had the newly revised GBV register. While most hospitals had PRC forms (83%) and SGBV registers (75%), these reporting tools were available in only 55% of health centers and less than one-third of dispensaries/clinics (27% for PRC forms and 20% for SGBV registers). Three-fourths of hospitals (75%) reported ability to conduct forensic medical examination for a survivor on the date of the assessment, compared with about one-third of health centers (32%) and dispensaries/clinics (31%). Overall, just 16% of facilities had secure storage, such as a lockable metal box or steel locker cabinet, for preservation of GBV-related medical evidence (dispensaries/clinics, 9%; health centers, 23%; hospitals, 50%).

**TABLE 4.** Readiness to Provide Components of Minimum Care Package for Sexual Violence on the Day of the Assessment Among Health Facilities Providing Routine GBV Care, by Tier of Care, Kenya, No. (%)

**Variables**	**Dispensary/Clinic (Tier 2)** **N=71**	**Health Center (Tier 3)** **N=22**	**Hospital (Tiers 4 and 5)** **N=12**	**Total (All Tiers)** **N=105**
**Information collection, documentation, medical tests, and storage of forensic samples – Availability of: **				
Forensic medical examination services	22 (31.0)	7 (31.8)	9 (75.0)	38 (36.2)
MOH reporting tools				
PRC forms	19 (27.1)	12 (54.6)	10 (83.3)	41 (39.4)
SGBV registers	14 (19.7)	12 (54.6)	9 (75.0)	35 (33.3)
Medical tests				
Pregnancy diagnostic test^a^	31 (77.5)	19 (90.5)	11 (100)	61 (84.7)
HIV test^a^	28 (70.0)	20 (95.2)	10 (90.9)	58 (80.6)
Syphilis or VDRL test	28 (39.4)	17 (77.3)	11 (91.7)	56 (53.3)
Urinalysis	23 (32.4)	18 (81.8)	11 (91.7)	52 (49.5)
High vaginal swab test	12 (16.9)	7 (31.8)	11 (91.7)	30 (28.6)
Hemoglobin test	17 (23.9)	10 (45.5)	11 (91.7)	38 (36.2)
Safe custody facility for GBV evidence	6 (8.5)	5 (22.7)	6 (50.0)	17 (16.2)
**Availability of emergency medical treatments and care: **				
HIV prevention				
PEP for adult	33 (46.5)	19 (86.4)	10 (83.3)	62 (59.1)
PEP for children	14 (19.7)	15 (68.2)	10 (83.3)	39 (37.1)
Pregnancy prevention				
Levonorgestrel (1.5 mg or 0.75 mg) pill	12 (16.9)	2 (9.1)	4 (33.3)	18 (17.1)
Combined oral contraceptive pill	63 (88.7)	16 (72.7)	10 (83.3)	89 (84.8)
Any emergency contraception product^b^	65 (91.6)	16 (72.7)	11 (91.7)	92 (87.6)
Post-exposure prophylactic vaccination				
Hepatitis B	13 (18.3)	3 (13.6)	3 (25.0)	19 (18.1)
Tetanus toxoid	65 (91.6)	22 (100)	11 (91.7)	98 (93.3)
STI prevention with antibiotics	46 (64.8)	17 (77.3)	11 (91.7)	74 (70.5)
Trauma counseling support	61 (85.9)	22 (100)	11 (91.7)	94 (89.5)

Abbreviations: GBV, gender-based violence; MOH, Ministry of Health; PEP, post-exposure prophylaxis for HIV with antiretroviral drugs; PRC, post-rape care; SGBV, sexual and gender-based violence; STI, sexually transmitted infection; VDRL, venereal disease research laboratory.

a Analysis excluded 33 facilities including 31 dispensaries/clinics, 1 health center, and 1 hospital that did not have a functional laboratory because the study did not inquire about the availability of point-of-care tests.

b Availability of emergency contraception products was defined as per the national guidelines, including the levonorgestrel pill (1.5 mg or 0.75 mg) or combined oral contraceptive pill.

Consistent with quantitative data, interviewed providers reported that stock-outs of violence reporting tools including PRC forms and SGBV registers were common, particularly among dispensaries/clinics and health centers, making proper documentation impossible. Several providers mentioned resorting to improvisation to capture GBV data by documenting it informally in other reporting tools, such as outpatient, family planning, or HIV registers; others simply recorded GBV data in a book.

*The MOH has not given us these reporting tools … we improvise just a big book whereby we report all cases of violence …* – Hospital 1, County B. Female Nurse

*… We have been capturing them in other tools like for diagnosis… We also capture it in the FP [family planning] register … in the HIV register … We need to have the reporting tools for GBV, it is a requirement.* – Dispensary/Clinic 3, County D, Female Nurse

Most facilities across all tiers had testing services for diagnosis of HIV infection (81%) and pregnancy (85%) on the day of the assessment (Table 4). However, a smaller percentage of dispensaries had urinalysis test (32%) and syphilis test (39%) than health centers (82% and 77%, respectively) and hospitals (92%, for both). While the availability for high vaginal swab and hemoglobin tests was low in dispensaries/clinics (17% and 24%, respectively) and health centers (32% and 46%, respectively), availability for both tests was high in hospitals (92%). Interviewed providers reported several gaps that affected provision of laboratory services. Many providers across all tiers of the health care system cited stock-out of reagents and lack of equipment for specimen collection as common challenges.

*Sometimes there are stock-outs of the reagents in the lab. Like now we don’t have Hb [hemoglobin test]. We cannot do it, and it is really big concern…* – Health Center 1, County B, Female Clinical Officer

In addition, several primary health care providers, in Tiers 2 and 3, reported there were no medical testing services in their facilities due to a complete lack of laboratory infrastructure. Other providers explained testing services were interrupted when laboratory staff was not available, mostly during the weekends and when on leave. Inability to provide essential laboratory services at the point of first contact with the survivors led to referral of survivors to higher-tier facilities for investigations and further management.

*My lab technician is not present because he has gone for a leave. There are some tests which cannot be done to the clients…* – Dispensary/Clinic 1, County C, Female Nurse

*We refer in case she [the survivor] comes on weekend … You assist where necessary and then you refer … to a hospital that has a functioning lab*. – Health Center 1, County D, Female Nurse

Most facilities had prophylactic and treatments for bacterial STIs (71%), the MOH-recommended emergency contraception product (88%), GBV-related counseling services (90%), and tetanus toxoid vaccines (93%) on the date of the assessment (Table 4). However, the availability of PEP medicines for HIV for children and adults was considerably lower among dispensaries/clinics (20% and 47%, respectively) than health centers (68% and 86%, respectively) and hospitals (83%, for both). Few facilities of any tier had hepatitis B vaccines (dispensaries/clinics, 18%; health centers, 14%; hospitals, 25%).

### Provider Knowledge

When presented with a case vignette describing a hypothetical case of an adolescent girl seeking post-rape care, most providers across all tiers of health care demonstrated correct theoretical knowledge for managing sexual violence, describing the minimum care package per national sexual violence guidelines. Providers also mentioned multiple components of person-centered care, such as ensuring privacy and confidentiality of services, administration of emergency therapies to prevent pregnancy and HIV, trauma counseling, and linkage to legal, shelter, and child protection services (Supplement Table).

*Welcome the patient to the facility, take her history, observe vital signs, and take her temperature to rule out infection. Take her pulse rate, blood pressure, and then do a physical exam from head and then move systematically to all body parts and finally examine her genitalia to observe/inspect if there is a tear or discharge in the vulva and then counsel her, test for HIV; if it is negative, start her on PEP. Treat soft tissue injuries in case of a tear and offer tetanus injection and give pain killers to relieve pain, offer emergency contraception, mostly we use COCs [combined oral contraceptives] to act as ECs [emergency contraceptives]. Inform them not to take a bath/shower, refer them to County Referral Hospital, record in the register [SGBV register] and ask the person who has brought the girl to go and report the incident to nearest police post/station. We will also do a follow up after 2 weeks.* – Dispensary/Clinic 2, County D, Female Nurse

… *Of course, safety is very important, yes. I have to assure this girl (the survivor) that this thing … I am going to be as secretive as much as possible. Okay, privacy is key … So, I will take that girl, I will bring her here [SGBV room] if I am not so busy, I will take her to this SGBV room*. – Hospital 1, County B, Female Nurse

Despite high levels of knowledge, many providers at primary health care facilities indicated differing clinical practices that constrained timeliness and quality of care mostly due to limited resources and prevalent stock-out of essential supplies. These challenges were cited to derail provision of timely and comprehensive services at many primary health care facilities and therefore contributed to upward referral of survivors.

*I will do counseling. I take samples all those forensic. Because maybe I take the urine. I take the gloves, I have to examine. Because I guess that lady had some struggle, so if the clothes were torn, I have to take those clothes, put it in a bag and then … in this place actually we are supposed to have some clothes [dignity kits] to change, so far, we don’t. So, I am giving you the real scenario what it is supposed to be.* – Hospital 1, County B, Female Nurse

*Number one, we have to receive this patient and we test for HIV infection … then we give prophylaxis [PEP]. We give emergency [contraceptive] pill. Then, because we don’t have a lot, we have to refer to higher level for further management.* – Health Center 2, County C, Male Nurse

In addition, providers highlighted gaps in the provision of psychosocial trauma counseling services to survivors due to lack or absence of qualified psychologists. Several providers reported that they were relying on other categories of counselors, particularly HIV testing counselors, to provide services even though they are not skilled to effectively manage the scope of trauma and mental health.

*… psychological counselor may not be available. So, there are cases whereby the only counselor available at the facility is the HTS [HIV Testing Services] who strictly deals with HIV counseling and testing … they are not really trained on giving other counseling services.* – Hospital 2, County B, Female Medical Doctor

### Availability of Charts and Abstracted Data

While clinical chart reviews were conducted in all 123 study facilities, over three-fourths of facilities (82%, n=101/123) did not have any charts available (data not shown). Nearly all dispensaries/clinics (94%, n=81/86) and over two-thirds of health centers (71%, n=17/24) had no documented charts, compared with approximately one-fourth (23%, n=3/13) of the study hospitals. Overall, 285 clinical charts were abstracted, which included 177 (62%) from hospitals, 76 (27%) from health centers, and just 32 (11%) from dispensaries/clinics (Table 5).

**TABLE 5.** Description of Survivors and Aggressors of Sexual Violence, Characteristics of Violence, and Reporting Patterns Based on Abstracted Chart Data, by Tier of Care, Kenya, No. (%)

**Variables**	**Dispensary/Clinic (Tier 2)** **N=32**	**Health Center (Tier 3)** **N=76**	**Hospital (Tiers 4 and 5)** **N=177**	**Total (All Tiers)** **N=285**
**Abstracted charts by county**				
Garissa	0 (0)	0 (0)	33 (18.6)	33 (11.6)
Kwale	32 (100.0)	58 (76.3)	85 (48.0)	175 (61.4)
Narok	0 (0)	17 (22.4)	38 (21.5)	55 (19.3)
West Pokot	0 (0)	1 (1.3)	21 (11.9)	22 (7.7)
**Survivors’ demographics data**				
Age in years				
<18 years	21 (65.6)	54 (72.0)	109 (68.6)	184 (69.2)
18 years or above	11 (34.4)	21 (28.0)	50 (31.4)	82 (30.8)
Missing value^a^	0	1	18	19
Gender				
Female	32 (100)	71 (94.7)	174 (98.3)	277 (97.5)
Male	0 (0)	4 (5.3)	3 (1.7)	7 (2.5)
Missing value^a^	0	1	0	1
Marital status				
Single	27 (84.4)	56 (77.8)	145 (86.3)	228 (83.8)
Married	5 (15.6)	9 (12.5)	20 (11.9)	34 (12.5)
Divorced/widowed/separated	0 (0)	7 (9.7)	3 (1.8)	10 (3.7)
Missing value^a^	0	4	9	13
**Characteristics of sexual violence**				
Aggressor known to survivor				
Known	14 (58.3)	43 (79.6)	108 (77.7)	165 (76.0)
Unknown	10 (41.7)	11 (20.4)	31 (22.3)	52 (24.0)
Missing value^a^	8	22	38	68
Relationship of aggressor to survivor among survivors who knew their aggressor	**N=14**	**N=43**	**N=108**	**N=165**
Partner (current or former)	1 (7.1)	25 (59.5)	26 (34.7)	52 (39.7)
Neighbor	11 (78.6)	13 (31.0)	24 (32.0)	48 (36.6)
Relative	1 (7.1)	3 (7.1)	3 (4.0)	7 (5.3)
Friend	1 (7.1)	1 (2.4)	4 (5.3)	6 (4.6)
Other associations	0 (0)	0 (0)	5 (6.7)	5 (3.8)
No association	0 (0)	0 (0)	13 (17.3)	13 (9.9)
Missing value^a^	0	1	33	34
Type of sexual violence experienced				
Vaginal	12 (37.5)	26 (35.1)	135 (81.8)	173 (63.8)
Anal	0 (0)	1 (1.4)	3 (1.8)	4 (1.5)
Combined (vaginal, anal, or oral)	1 (3.1)	1 (1.4)	3 (1.8)	5 (1.8)
Unspecified penetration	18 (56.3)	36 (48.6)	15 (9.1)	69 (25.5)
Non-penetrative	1 (3.1)	10 (13.5)	9 (5.5)	20 (7.4)
Missing value^a^	0	2	12	14
**Violence reporting and care-seeking**				
Reported to police before presenting at the current facility				
Yes	5 (71.4)	30 (93.8)	117 (95.9)	152 (94.4)
No	2 (28.6)	2 (6.3)	5 (4.1)	9 (5.6)
Missing value^a^	25	44	55	124
Timing of health care-seeking after violence, among survivors that presented at the health facility for the first time^b^	**N=31**	**N=72**	**N=157**	**N=259**
Same day	17 (63.0)	26 (38.8)	11 (10.2)	54 (26.7)
2–3 days	3 (11.1)	15 (22.4)	46 (42.6)	64 (31.7)
4–5 days	0 (0)	3 (4.5)	6 (5.6)	9 (4.5)
6 or more days	7 (25.9)	23 (34.3)	45 (41.7)	75 (37.1)
Missing value^a^	4	4	49	57

^a^ Missing data are excluded when calculating proportions.

^b^ Denominator excluded 26 observations that had previously sought facility-based care, including 1 in a dispensary/clinic, 5 in a health center, and 20 in a hospital.

Interviewed providers indicated some facilities had no sexual violence charts available for abstraction because there were no survivors that presented for care, while other facilities were unable to document cases due to lack of reporting tools. Conversely, when reporting tools were available, several providers revealed that facility data were captured inconsistently and were therefore incomplete. Providers mentioned challenges related to human resources such as lack of GBV-trained staff, limited time available for documentation due to high patient workload and lengthy GBV documentation process, and perceived providers’ negative attitudes that contributed to poor case recording and documentation.

*… in case it is a rape it needs a lot of time to fill the forms, screening, and everything … We are 2 officers so in case one has gone for annual leave you are left alone here, and you have outpatients for you to attend, you have CCC [HIV clinic] for you to attend, you have MCH [maternal and child health] for you to attend …* – Health Center 2, County B, Male Clinical Officer

### Survivors’ Characteristics

Among reviewed charts, most sexual violence survivors were children aged less than 18 years old (69%), female (98%), and unmarried/single (84%) (Table 5). Seventy-six percent of aggressors were known to the survivors, with the most frequently identified as either a current/former intimate partner (40%) or a neighbor (37%), with a smaller proportion identified as relatives (5%) or friends (5%). Most charts documented penetrative sexual violence (vaginal, 64% and unspecified penetration, 26%).

### Perspectives on Violence Reporting and Care-Seeking

Reviewed charts showed that almost all occurrences (94%) of sexual violence had already been reported to the police before survivors presented for health care services at the current facility (Table 5). Approximately two-thirds of survivors presented at a health facility for the first time within the recommended 72-hour window period (27% on the same day, and 32% between 2–3 days). Providers held different beliefs about the primary barriers to timely care-seeking among survivors, with some mentioning fear, social stigma, and lack of awareness about health services, while others mentioned distance to health facilities and lack of transport.

*Mostly because of fear of society or the norms of society, they don’t come to get services***.** – Health Center 7, County A, Female Nurse

*Number one is accessibility and transport services because somebody cannot be like in … 100 kilometers away and was physically assaulted and travel all the way to [named a town] …* – Dispensary/Clinic 1, County D, Male Clinical Officer

Qualitative interviews highlighted examples of sexual violence that would not be captured through chart reviews, such as survivors who report that they were raped only when presenting at the health facility for antenatal care or labor and delivery.

*They don’t report to the facility early because of that stigma especially when it happens in their own homes … Most of them come when they are already pregnant or maybe they have delivered is when they will tell you “I was raped” …* – Hospital 1, County B, Female Nurse

The overwhelming majority of providers also mentioned that many survivors sought assistance through community and law enforcement structures, but they did not always accompany this by seeking medical care. While the police station and chief were stated as key administrative pathways for GBV reporting, many providers believed that many sexual violence cases were being settled informally through traditional criminal justice systems.

*Most of them run to the parents or “wazee wa mtaa” [village elders] or the chief … they just cool it down until you realize there is pregnancy, that’s when the parent can take action now. When they go to the village chairman, they force the child to say who the pregnancy is [from]. If they say, they call that person, they do it under water [secretly] … That’s why you see a lot of teenage pregnancy here … There is what we call Kangaroo courts, they solve underground, they don’t bring to the hospital.* – Hospital 1, County B, Female Nurse

However, providers opined that many survivors sought facility-based care when violence-related injuries were perceived to be serious and life-threatening, a repeated violation, or when sexual violence resulted in a pregnancy, particularly among teenagers. Providers emphasized beliefs that cases seen by the facilities are just a small proportion of all sexual violence, representing only the cases that are deemed as the most severe or serious by the community.

*… they report to law enforcement officer and police those things [GBV] and in case they are really injured they seek for health services … when somebody is seriously injured and bleeds …* – Health Center 2, County A, Male Nurse

*The data that is collected from the facility, it is fewer compared with those who go to report to the gender-based violence desk at the police stations, or the cases that are reported to the chiefs.* – Dispensary/Clinic 3, County D, Female Nurse

### Quality of Care

Approximately one-half of eligible sexual violence survivors across all tiers of the health care system received emergency contraception (dispensaries/clinics, 47%; health centers, 46%; hospitals, 58%) (Table 6). Similarly, about one-half of eligible survivors received PEP for HIV (dispensaries/clinics, 58%; health centers; 53%; hospitals, 51%) and antibiotic treatment for STI management (dispensaries/clinics, 53%; health centers, 57%; hospitals, 57%). Documentation to indicate completion of psychosocial assessment was consistently low at all tiers of health care system (dispensaries/clinics, 32%; health centers, 16%; hospitals, 20%). Overall, a substantial proportion of eligible survivors failed to receive some components of facility-based care, including psychosocial assessment (32%), PEP for HIV (22%), emergency contraception (17%), and antibiotic treatment for STI management (15%). The research team also observed a relatively high amount of missing data within the reviewed charts: For documentation of specific components of care, between one-fourth (26%) and one-half (48%) of abstracted charts were missing data.

**TABLE 6.** Emergency Treatments and Care Provided to Eligible Survivors of Sexual Violence^a^ Based on Abstracted Chart Data, by Tier of Care, Kenya, No. (%)

	**Dispensary/Clinic** **(Tier 2)**	**Health Center** **(Tier 3)**	**Hospital** **(Tiers 4 and 5)**	**Total** **(All Tiers)**
Psychosocial assessment	**N=31**	**N=58**	**N=161**	**N=250**
Yes	10 (32.3)	9 (15.5)	32 (19.9)	51 (20.4)
No	0 (0)	15 (25.9)	64 (39.8)	79 (31.6)
Not documented (missing value)	21 (67.7)	34 (58.6)	65 (40.4)	120 (48.0)
PEP with antiretroviral viral drugs	**N=19**	**N=34**	**N=49**	**N=102**
Yes	11 (57.9)	18 (52.9)	25 (51.0)	54 (52.9)
No	2 (10.5)	7 (20.6)	13 (26.5)	22 (21.6)
Not documented (missing value)	6 (31.6)	9 (26.5)	11 (22.5)	26 (25.5)
Treatment for bacterial STIs	**N=19**	**N=35**	**N=51**	**N=105**
Yes	10 (52.6)	20 (57.1)	29 (56.9)	59 (56.2)
No	2 (10.5)	3 (8.6)	11 (21.6)	16 (15.2)
Not documented (missing value)	7 (36.8)	12 (34.3)	11 (21.6)	30 (28.6)
Emergency contraception pill	**N=15**	**N=28**	**N=38**	**N=81**
Yes	7 (46.7)	13 (46.4)	22 (57.9)	42 (51.9)
No	3 (20.0)	4 (14.3)	7 (18.4)	14 (17.3)
Not documented (missing value)	5 (33.3)	11 (39.3)	9 (23.7)	25 (30.9)

Abbreviations: PEP, post-exposure prophylaxis for HIV with antiretroviral drugs; STI, sexually transmitted infection.

^a^ Proportions are calculated based on a subset of survivors that were determined eligible for each treatment or care.

### Perspectives on Documentation Practices and Legal Services

Qualitative interviews with providers confirmed poor GBV documentation practices were common across all tiers of the health care system. Beyond lack of GBV training relevant to data reporting, providers opined that poor documentation practices were also deliberate on the part of providers who wished to avoid providing any documentation that would require them to attend court hearings to present medical evidence. Providers reported that this reluctance was due to lack of logistical support when court hearings were required. Other providers felt that lack of medico-legal skills was causing fear and anxiety when presenting medical reports in the courts.

*… you fill that form, and you are needed to go and defend that case and you do not have transport, and you’ll hear a provider saying that I will not fill the PRC form. And am referring this client [survivor] because I won't handle that case*. – Health Center 4, County C, Female Nurse

*… fear of going to the court is a major problem with these guys [providers] here … Everybody doesn’t want to help, and you know these things is for nurses, clinical officers, medical officers, for any health care worker who gives the services you have to chip in … A patient can only be taken in round in circles for a whole week. I have seen many scenarios, a patient comes. We are not filling P3 on Monday, go up to Wednesday just because you don’t want to fill it …* – Hospital 1, County B, Female Nurse

### Provider Attitudes

Many interviewed providers believed that if supported by the health care system, many providers would have positive attitudes toward management of sexual violence, including facilitating access to legal justice. Specifically, providers felt that strategic interventions were required to motivate frontline providers by ensuring they received relevant GBV training, continuous availability of supplies in the facilities, and ways to address logistical challenges when providers are required to bring forth medical evidence in the courts.

*… they have good attitude they are willing to manage all the client as long as health care worker has enough knowledge about that case … they are ready to help this client, but medicine is dynamic every day …* – Health Center 6, County A, Male Clinical Officer

Positively, several providers reported going above and beyond the call of duty including committing personal time and expending financial resources that ensured survivors accessed justice in processes that were long and drawn-out.

*… the case dragged itself for 4 years, and I had to spend my time and money. All that time, I was going to give my testimonies … when you assist a patient, and sometimes you literally waive the costs for the patient, because they might not have money, and you treat them … It is very discouraging sometimes, but we do our best to make sure we do the right thing.* – Dispensary/Clinic 3, County D, Female Nurse

## DISCUSSION

This study examined readiness for facility-based health care services, provider experiences, and quality of medical care provided to survivors of sexual violence. Although national guidelines stipulate all tiers of the Kenyan health care system—from dispensaries to referral hospitals—should offer a minimum package of sexual violence services,^24^ this study reveals several critical gaps that should be considered in further strengthening the health care system’s response to sexual violence.

We observed steep gradients in sexual violence service readiness across tiers of the Kenyan health care system. Fewer than half of dispensaries and clinics were able to provide essential services such as routine PEP for HIV (for either adults or children), post-assault forensic medical examination, or basic laboratory services. Dispensaries and clinics are often the first point of care-seeking, particularly for Kenya’s rural communities;^26^ however, a review of facility data showed sexual violence was seldomly managed at the dispensary/clinic level. We found that approximately a fifth of study dispensaries/clinics did not offer GBV services, and there were no clinical charts with survivors’ data available for a period of 6 months preceding the date of the assessment in more than 90% of dispensaries/clinics. Inability to deliver essential services at these first-line facilities requires survivors to seek care at higher-level facilities—a barrier with substantial time and out-of-pocket costs borne solely by survivors. In the case of time-sensitive treatment protocols, additional barriers to care that result in delays can have major deleterious health consequences for survivors including, but not limited to, HIV and other STIs and unwanted pregnancy in a setting where abortion is highly restricted.^27^^,^^28^ A key implication of these findings is the need to strengthen the health care system’s ability to provide a minimum emergency package of sexual violence care within primary health care facilities—eliminating the need for survivor transfer—and to strengthen effective and timely referral systems when specialized care is required.

We also observed missed opportunities for sexual violence treatment even among survivors who arrived promptly for medical care. Concerningly, we estimated one-fifth to one-third of eligible survivors were not initiated with emergency therapies to prevent pregnancy, HIV, and bacterial STIs, or offered psychosocial service to address trauma. Similar findings were reported in recent studies in Kenya, in which 24% of eligible sexual assault survivors were not given HIV PEP,^16^ 50% were not given emergency contraception, and 20% were not given STI treatment.^29^ At a systems level, our qualitative insights suggest that a lack of a critical mass of GBV-trained providers coupled with shortages of essential health care supplies drive noted gaps in the management of sexual violence cases. While there was moderate to high facility availability of several emergency medical treatments, the availability of hepatitis B vaccination was notably low across all tiers of the health care system (14% to 25%). Inability to provide post-exposure vaccination to prevent hepatitis B infection to survivors of sexual violence, including at referral hospitals, is particularly concerning considering hepatitis B is a major public health problem in Kenya, with an estimated prevalence of more than 8% in the general population,^30^ and an emerging leading cause of long-term health challenges, including liver damage, liver failure, liver cancer, disability, and death.^31^^,^^32^

Our results also highlight systemic challenges with health facility documentation of sexual violence, including the low availability and use of post-rape care forms and national facility-based documentation tools. Even when reporting tools were available in the facility, our findings suggest that documentation was often incomplete and of low quality, making quality of care assessment challenging or impossible. This finding has several implications: at the individual level, lack of use of post-rape care forms—which are legally required for documentation of sexual violence and used as evidence in the legal system—means that survivors seeking care at facilities that do not stock or use these forms may lack critical medical evidence needed to seek legal recourse. In addition, poor routine documentation practices including missing data prevent monitoring of sexual violence caseloads and care quality at a systems level, thereby limiting evidence-based decision making in programming and resource allocation. Similar to previous studies, a dominant theme that emerged from our qualitative data revealed that health care providers were reluctant notetakers in matters related to sexual violence as they felt unqualified to manage the complexity of legal processes or because they experience logistical problems when delivering legal evidence in the courts.^13^^,^^33^ Our findings highlight other structural factors, including heavy caseloads with poor staffing, lack of funding and logistical support, and low perceived competency, that drive poor documentation. This practice gap further emphasizes the importance of ensuring frontline responders, including those serving in primary health care facilities, are adequately supported to manage survivors of sexual violence across all age categories, including in-depth capacity building in medico-legal aspects for improved chain of custody of evidence.

Our findings also illustrate that health care system strengthening strategies alone are not a panacea for improving survivors’ health and well-being outcomes. Consistent with quantitative data, a dominant theme that emerged across most interviews revealed that many survivors were unable to present for health care services within the critical 72-hour period to receive the most effective treatment and support. Similar findings were reported in recent study conducted in a Kenyan public hospital, in which more than 40% of sexual violence survivors presented too late to receive emergency therapies.^34^ Providers felt that survivors faced multiple barriers to care-seeking, which leads to delays including social stigmatization, lack of awareness about health care services, and navigating logistical challenges in reaching a skilled GBV provider who may be located far. While the Kenyan Sexual Offences Act of 2006 explicitly prohibits traditional justice systems from adjudicating sexual crimes,^35^ we found that these informal systems were commonly used as the first-line recourse for reporting and resolving sexual violence among many survivors. As many other studies have reported, informal justice systems are infamously known to obstruct justice to conceal the aggressor and in so doing may prevent or substantially delay health care seeking.^12^^,^^36^ There is urgent need to strengthen grassroots GBV prevention and response systems including community awareness campaigns and forming stronger linkages between community groups and health care facilities to improve health care-seeking behaviors related to sexual violence. To address some of the identified gaps, the *Accelerate* program was adapted to integrate a new workstream that supports strengthening of community-based shelter services across all 13 program counties. Beyond facilitating linkage to health care and legal services, shelters will be supported to provide other essential survivor-centered services including mental health support and skills-building to enhance coping, resilience, and livelihood empowerment.

## Limitations

This study has several limitations. While chart abstraction provides insights into the quality of care delivered to survivors of sexual violence, results from chart abstraction are subject to several forms of selection bias. First, charts represent only those survivors who presented at the health facility for care. Our qualitative results highlight that many cases, particular those deemed to be less “serious” by the local communities, such as teenage pregnancy or child marriage by an adult intimate partner, are rarely reported at the health facility. Second, many charts are incomplete, with poor documentation sometimes deliberate on the part of providers who do not want to represent their clients in court. While missing data is an important finding in and of itself, it limits our ability to verify actual quality of care. Results from chart abstraction should be interpreted cautiously to represent only those cases with higher-quality documentation; it is plausible that they overestimate care quality, as it is expected that more experienced providers at better resourced facilities would be more likely to complete documentation. Second, while all program-supported facilities were included in the sample, there were low numbers of private health facilities, which prevented meaningful sub-population analyses by sector. Third, our study facilities were not sampled to be nationally representative, so extrapolation of findings should be made with caution. Finally, participating health facilities had already received some early direct support from the *Accelerate* program at the time of data collection, meaning that findings may overestimate quality of care relative to a true pre-intervention baseline assessment.

## CONCLUSION

This study contributes to the sparse literature on provision and quality of sexual violence medical care in Kenya by identifying critical health care systems’ strengths and structural gaps. Given the observed steep gradients in sexual violence service readiness across tiers of the health care system, we advocate for increased investment in capacity-strengthening interventions that deliberately target primary health care facilities to provide a minimum package of essential services for sexual violence. We also advocate strong community safety partnerships to drive social norms shifts related to GBV prevention and response, including stronger linkages between communities and health facilities.

## Notes

**Peer Reviewed**

**First Published Online:** June 24, 2026.
